# Plankton Food Web Responses to Experimental Nutrient Additions in a Subtropical Lake

**DOI:** 10.1100/tsw.2006.176

**Published:** 2006-07-18

**Authors:** Karl E. Havens, Therese L. East

**Affiliations:** ^1^ University of Florida, Department of Fisheries and Aquatic Sciences, Gainesville, FL, USA; ^2^ South Florida Water Management District, West Palm Beach, FL, USA

**Keywords:** plankton, food webs, nutrients, cyanobacteria, subtropical lake

## Abstract

During two controlled enclosure experiments using water from a subtropical lake, the plankton food web displayed a highly variable response to combined addition of nitrogen and phosphorus. In July, the nutrients stimulated growth of *Cylindrospermopsis raciborskii*, and the biomass of macrozooplankton and microbial food web components did not increase. In October, the same addition of nutrients stimulated growth of small edible *Lyngbya* spp., and there were coincident increases in biomass of macrozooplankton and components of the microbial web. Past generalizations that cyanobacteria blooms inhibit growth of other food web components may not always hold true.

## INTRODUCTION

More than 35 years ago, Gliwicz[1] postulated that biomass partitioning in the plankton is substantively different in highly eutrophic lakes than in their less-enriched counterparts. He presented a conceptual diagram indicating that in eutrophic lakes, a considerable portion of phytoplankton biomass accrues in large inedible taxa, and that the major basal resource for higher trophic levels is bacteria. Since that time, there have been many papers describing what became known as the microbial web, which transfers dissolved organic carbon from bacteria to macrozooplankton (copepods and/or cladocerans) by way of nanoflagellates, ciliates, and/or microzooplankton such as rotifers[2,3]. Empirical data support the view that with increasing eutrophication, there is an increase in the relative importance of microbial pathways, an increase in the number of links in food webs connecting basal resources with macrozooplankton, and reduced food web efficiency[4]. This situation is acute in eutrophic subtropical lakes, where cooccurrence of small zooplankton and large phytoplankton may result in very low transfer efficiency in phytoplankton-based food webs and relatively greater carbon transfer *via* microbial webs[5,6,7].

This study expands on earlier observational work by experimentally testing the hypothesis that nutrient enrichment in a subtropical lake has predictable effects on plankton structure and function, including increased biomass of inedible net phytoplankton and a decline in macrozooplankton biomass.

## METHODS

### Study Site and Experimental Design

The experiments were conducted in July and October 2002 (early and late summer), using water and plankton collected from Lake Okeechobee, a large eutrophic lake located at 26$^o$ 58’ N 80$^o$ 50’ W in Florida. Detailed information regarding this ecosystem, including its plankton food web, is provided in Work et al.[7].

Water was collected with a submersible pump from approximately 2 km offshore from the south end of the lake (see Table 1 for physical, chemical, and biological conditions), and pumped into eight 20-l clear plastic carboys. Two treatments were established with quadruplicate replication: Controls (no nutrients added) and NP (N added at 2,000 μg $l^{-1}$ as $NaNO_3$ and P added at 200 μg $l^{-1}$ as $KH_2PO_4$). The 10:1 ratio of TN:TP (by mass) in the NP treatment was intended to facilitate dominance by cyanobacteria, which bloom in this lake under high irradiance, high P, low TN:TP conditions[8,9]. The carboys were placed inside large flow-through tanks located on the lake shore and held at a depth of approximately 0.5 m for 6 days with water temperatures approximating the adjacent lake.

**TABLE 1**
**Physical, Chemical, and Biological Conditions in the Lake at the Time when Water and Plankton Samples were Collected for the 2002 Food Web Experiments**

| Parameter | Units | July | October |
|---|---|---|---|
| Depth | cm | 160 | 200 |
| Secchi depth | cm | 120 | 55 |
| Temperature (mid-depth) | $^oC$ | 29 | 28 |
| Total phytoplankton | $\mu m^3\ ml^{-1}$ | $899 \times 10^6$ | $2 \times 10^6$ |
| Total macrozooplankton | $\mu g\ l^{-1}$ | 8 | 12 |
| Dominant phytoplankton | | *Anabaena* | *Aulacoseira*, *Lyngbya* |
| Dominant zooplankton | | *Diaptomus dorsalis* | *D. dorsalis* |

### Plankton Sampling and Biomass Measurement

Following incubation, the carboys were retrieved under a shaded structure to avoid exposure to direct sunlight and samples were collected to determine the carbon biomass of bacteria-plankton, picoplankton, autotrophic and heterotrophic nanoplankton, net phytoplankton (>20 μm), ciliates, rotifers, and macrozooplankton. Samples were enumerated in the laboratory using a light microscope and epifluorescent methods[10,11,12]. Species and community carbon biomass values were determined from published relationships with body size, dry weight, or biovolume: Scavia and Laird[13] for bacteria and picoplankton; Putt and Stoecker[14] for ciliates; Strathmann[15] for nanoplankton and net phytoplankton, and Anderssen and Hessen[16] for zooplankton.

## RESULTS

In July 2002, net phytoplankton dominated carbon biomass in the food web (Fig. 1) and addition of nutrients led to increased biomass of picoplankton, phototrophic nanoflagellates, and net phytoplankton. None of the microbial components of the food web displayed increased biomass, nor did the zooplankton.

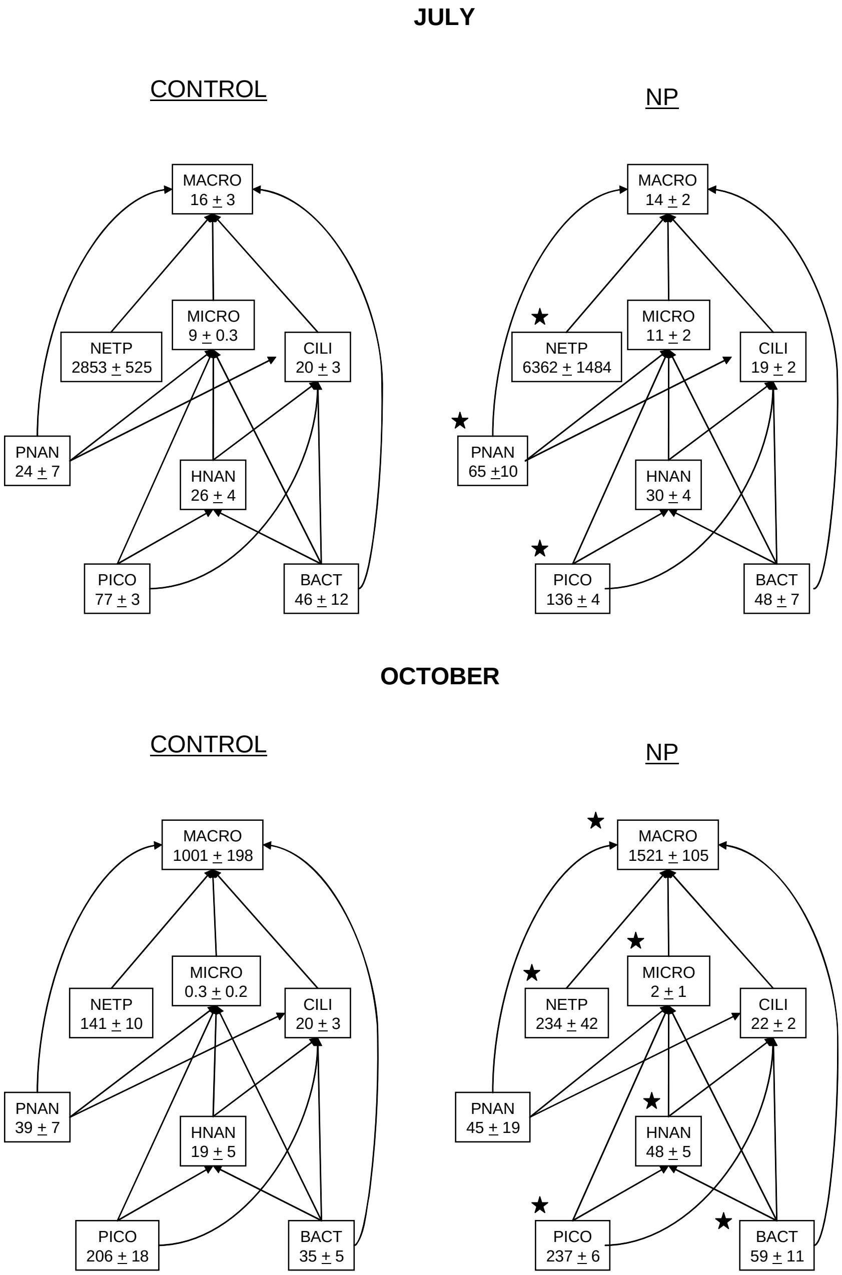

**FIGURE 1.** Carbon biomass in various components of the plankton food web after 6 days of incubation in control (C) and nutrient addition (NP) treatments described in the text. Numbers are carbon biomass in μgC $l^{-1}$ and arrows connecting boxes (not quantified) represent known pathways of carbon flux in the food web of this lake[7]. Acronyms are: MACRO = macrozooplankton, MICRO = microzooplankton, NETP = net phytoplankton, CILI = ciliates, PNAN = phototrophic nanoflagellates, HNAN = heterotrophic nanoflagellates, PICO = phototrophic picoplankton, and BACT = bacteria plankton. Stars indicate significant differences between treatments based on a one-way ANOVA with $p < 0.05$. Values are means ± one standard deviation.

Taxonomic analysis indicated that the increased biomass of net phytoplankton was caused by a bloom of filamentous cyanobacteria, which accounted for nearly 100% of biomass in that plankton category in both the control and NP treatments. Importantly, nutrient addition shifted the composition of the phytoplankton from dominance by short filaments of *Lyngbya limnetica* to larger filaments of *Cylindrospermopsis raciborskii* (Table 2). The zooplankton was dominated by one calanoid copepod, *Diaptomus dorsalis*, and one cyclopoid copepod, *Acanthocyclops vernalis*, and their absolute and relative biomass values were not affected by nutrient addition (Table 3).

**TABLE 2**
**Percent of Total Biomass of the Dominant (>10% Total) Microphytoplankton in the Control (C) and NP Treatments of the July and October 2002 Food Web Experiments (Values are Means ± One Standard Deviation)**

| Month | Taxa | C | NP |
|---|---|---|---|
| July | *L. limnetica* | 41 ± 8 | 25 ± 4 |
| | *Anabaena* spp. | 23 ± 9 | 25 ± 4 |
| | *C. raciborskii* | 3 ± 4 | 40 ± 9 |
| October | *Aulacoseira granulata* | 40 ± 7 | 0 |
| | *C. raciborskii* | 15 ± 3 | 0 |
| | *L. contorta* | 16 ± 1 | 16 ± 2 |
| | *L. limnetica* | 10 ± 1 | 34 ± 4 |
| | *Anabaena* spp. | 6 ± 1 | 21 ± 3 |

**TABLE 3**
**Percent of Total Biomass of the Dominant (>10% Total) Macrozooplankton in the Control (C) and NP Treatments of the July and October 2002 Food Web Experiments (Values are Means ± One Standard Deviation)**

| Month | Taxa | C | NP |
|---|---|---|---|
| July | *D. dorsalis* | 47 ± 10 | 55 ± 5 |
| | *Acanthocyclops vernalis* | 35 ± 1 | 34 ± 9 |
| October | *D. dorsalis* | 70 ± 11 | 74 ± 11 |
| | *Mesocyclops edax* | 29 ± 3 | 24 ± 11 |

In October, the majority of carbon biomass occurred in macrozooplankton (Fig. 1) and net phytoplankton biomass was much lower than in July. Nutrient addition resulted in increased biomass of picoplankton, heterotrophic nanoflagellates, net phytoplankton, microzooplankton (rotifers and nauplii), and macrozooplankton. On this occasion, the net phytoplankton was dominated by *Aulacoseira* (a diatom) and *Lyngbya*, and nutrient addition stimulated growth of the latter alga. *C. raciborskii* did not bloom in this experiment; in fact, it declined to low levels in the NP treatment.

## DISCUSSION

The concept of predictable plankton responses to increased nutrient inputs was not supported by this experiment. Rather, the results indicate that responses of subtropical plankton to nutrient enrichment can vary considerably, depending on genus-level responses in the cyanobacteria assemblage. This finding has important implications for efforts to predict the response of food webs to changes in nutrient loading.

When lakes experience increased nutrient enrichment and associated symptoms, including cyanobacterial blooms, observational data indicate a tendency for the biomass of plankton to become more focused in net phytoplankton[17], and for the ratio of zooplankton to phytoplankton biomass to decline due to a predominance of large inedible algae[18]. Filamentous and colonial cyanobacteria that dominate in highly eutrophic lakes have been shown to reduce the grazing activities of crustacean zooplankton, either through mechanical interference or by various effects of toxins[19]. Yet most studies to examine effects of high cyanobacteria biomass on macrozooplankton have been done with cladocerans, in particular with large species of *Daphnia*. These grazers play an important role in controlling algal biomass in some temperate lakes, at times even producing spring clear water phases[20], and their biomass and degree of influence on phytoplankton do predictably decline in late summer when cyanobacteria blooms occur. Lakes in the subtropics differ from this scenario in that (1) phytoplankton seasonal dynamics are less regular and (2) the lakes do not support large-bodied *Daphnia*, perhaps due to intense fish predation. Lake Okeechobee is typical of eutrophic Florida lakes in having a zooplankton assemblage dominated by calanoid copepods and rotifers, along with lower densities of small cladocerans such as *Eubosmina*, *Bosmina*, and *Chydorus*[21].

The results of this study indicate that subtropical macrozooplankton can display a positive response to nutrient addition, and that this response depends on the taxonomic composition of the cyanobacteria. In the July experiment, when the phytoplankton increase was associated with a bloom of *C. raciborskii*, macrozooplankton biomass did not respond, whereas in October, when the phytoplankton increase was due to *Lyngbya* spp., macrozooplankton biomass increased by 50%. We previously have shown[22] that *Diaptomus dorsalis* and the other dominant macrozooplankton taxa in Lake Okeechobee consume small filamentous cyanobacteria, including *Lyngbya* and *Oscillatoria*. The dominant *Lyngbya* spp. occur as short filaments (five to eight cells) that probably do not interfere with filtering activities and are readily grazed from the water. There are no reports of toxin production associated with *L. contorta* or *L. limnetica* in Florida. Thus, when nutrients stimulated algal growth in October, they provided an enhanced food resource for the macrozooplankton, which in turn displayed increased biomass. Likewise, the macrozooplankton may have benefited from increased carbon flow in microbial pathways, where bacteria, heterotrophic nanoflagellates, and microzooplankton all increased in the October experiment. From gut analysis and studies of carbon transfer, we previously have shown that *D. dorsalis* directly grazes bacteria, as well as feeding on the larger components of the microbial web[7,22].

In contrast, nutrients stimulated a bloom of *C. raciborskii* in the July experiment and macrozooplankton biomass did not increase. There are several explanations for this result that should be followed up in future research, given the paucity of information about how this common cyanobacterium affects other components of the plankton. First, it might be that the larger size of *C. raciborskii* filaments resulted in mechanical interference with grazing by the copepods, as occurs when *Daphnia* grazes on other filamentous taxa such as *Anabaena*[23]. One problem with this explanation is that prior studies of copepod grazing on filaments[24] indicate that filaments may be more effectively grazed than unicells. Those authors concluded that *Diaptomus sicilis* had a “perceptual bias for capture of elongated algae.” However, the algae in that study were filaments of diatoms, so it is unclear whether they can be generalized to cyanobacteria. Another explanation is that macrozooplankton does not graze *C. raciborskii* due to toxicity because this alga produces the hepatotoxin cylindrospermopsin. The South Florida Water Management District has recorded low levels of this toxin in surface water samples collected from Lake Okeechobee in a routine algal monitoring program. We did not assay for toxins in this study, but it is reasonable to conclude that they were present, given the high biomass of the *C. raciborskii* that developed in the July experiment. Grazing and growth inhibition of crustacean zooplankton by cyanobacterial toxins, including those produced by *Anabaena, Aphanizomenon,* and *Microcystis,* are well established in the literature[25,26,27,28], but the effects of cylindrospermopsin have not been described.

Another explanation for the lack of macrozooplankton response to nutrients in July relates to the microbial web, whose components did not respond with increased biomass as they did in October. The reason for this is unclear. Only a small number of studies have considered nutrient effects on microbial food webs[29,30,31] and these studies have focused on humic lakes and marine systems. One possibility is that the microbial components were negatively affected by allelopathic compounds produced by *C. raciborskii*.

Finally, we cannot rule out the possibility that in July, conditions generally were not favorable for growth of macrozooplankton. Macrozooplankton biomass was two orders of magnitude higher in October than in July in the controls, so the experiments may have coincided with times of slow and fast growth of copepods in the lake. This could stem in part from filtering inhibition in July at very high algal biomass vs. absence of such an effect in October, a phenomenon documented nearly 40 years ago in feeding studies with *Diaptomus*[32]. Regardless of the explanation, the results of this study indicate that it is not always the case that eutrophic lakes display direct effect of added nutrients on biomass of net phytoplankton. Under certain conditions, added nutrients may be transported effectively to the macrozooplankton, by algal and/or bacterial based food webs, even when cyanobacteria strongly dominate the phytoplankton.

## ACKNOWLEDGMENTS

This work was supported by the South Florida Water Management District. The authors thank Andrew Rodusky for assistance in the field and laboratory, and Edward J. Phlips (University of Florida) and two anonymous reviewers for their constructive comments on an earlier version of this manuscript.
